# Negative Impact of Skeletal Muscle Wasting After Neoadjuvant Chemotherapy Followed by Surgery on Survival for Patients with Thoracic Esophageal Cancer

**DOI:** 10.1245/s10434-017-6020-2

**Published:** 2017-08-31

**Authors:** Shuhei Mayanagi, Yasuhiro Tsubosa, Katsuhiro Omae, Masahiro Niihara, Tsuneyuki Uchida, Takahiro Tsushima, Tomoya Yokota, Hiroshi Sato, Tateaki Naito, Hirofumi Yasui

**Affiliations:** 1 Division of Esophageal Surgery, Shizuoka Cancer Center Hospital, Shizuoka, Japan; 2 Clinical Research Promotion Unit of Clinical Research Center, Shizuoka Cancer Center, Shizuoka, Japan; 3 Division of Gastrointestinal Oncology, Shizuoka Cancer Center Hospital, Shizuoka, Japan; 4 Department of Esophagogastric Surgery, Saitama Medical University International Medical Center, Saitama, Japan; 5 Division of Thoracic Oncology, Shizuoka Cancer Center Hospital, Shizuoka, Japan

## Abstract

**Background:**

Skeletal muscle wasting during curative treatment is an important issue faced by esophageal cancer patients. However, it has not been clarified whether skeletal muscle change during neoadjuvant chemotherapy followed by surgery adversely affects prognosis. This study aimed to determine the relation between skeletal muscle change and survival for patients with advanced esophageal cancer who underwent neoadjuvant chemotherapy followed by surgery.

**Methods:**

This study retrospectively analyzed 66 patients with thoracic esophageal cancer who had undergone neoadjuvant chemotherapy followed by esophagectomy. The study investigated the correlation between the change in the total muscle cross-sectional area at the third lumbar vertebra before and 4 months after surgery as well as the postoperative recurrence and overall survival (OS).

**Results:**

Of the 66 patients, 39 (59%) showed a skeletal muscle decrease from baseline to 4 months after esophagectomy. The change in the skeletal muscle index from baseline to 4 months after surgery was −1.2 cm^2^/m^2^. Multivariable analysis showed that nonsquamous cell carcinoma subtype (hazard ratio [HR] 2.57; *p* = 0.029), pathologic stage (HR 5.73; *p* < 0.01), and skeletal muscle wasting (HR per 1 unit decrease in skeletal muscle index, 1.16; *p* = 0.015) were the independent prognostic factors associated with worse OS. Additionally, pathologic stage (HR 6.03; *p* < 0.01) and skeletal muscle wasting (HR per 1 unit decrease in skeletal muscle index, 1.11; *p* = 0.048) also were found to be independent prognostic factors associated with worse recurrence-free survival.

**Conclusions:**

The study findings suggest that skeletal muscle wasting from baseline has a negative impact on cancer recurrence and survival.

Thoracic esophageal cancer has a poor prognosis, especially in the advanced stage, and it is the sixth most frequent cause of cancer death worldwide.^1^ Patients with advanced esophageal cancer frequently have sarcopenia, defined as the loss of skeletal muscle mass, at diagnosis. Dysphagia and weight loss often result from malignant esophageal stenosis. Skeletal muscle loss related to cancer is the outcome of a negative energy and protein balance driven by a variable combination of reduced food intake and abnormal metabolism.^2^

Skeletal muscle loss is influenced not only by the nature of the esophageal cancer but also by the multimodality approaches for cancer treatment, including chemotherapy, radiotherapy, and surgery. Neoadjuvant chemoradiotherapy or chemotherapy followed by surgery has been selected as the standard treatment approach for advanced esophageal cancer.^3^,^4^ After curative surgery, patients often cannot orally ingest adequate amounts of food because of alimentary tract reconstruction using the gastric tube. Thus, multiple factors related to the primary esophageal cancer and to the invasive treatment for curative intent cause skeletal muscle loss.

Studies have shown that sarcopenia is associated with a poor prognosis in various cancers, including gastrointestinal^5^–^10^ and hepatopancreatobiliary malignancies.^11^–^14^ Although several retrospective studies analyzing sarcopenia in thoracic esophageal cancer patients have been published, it is controversial whether pretreatment sarcopenia results in a poor prognosis.^6^,^15^–^17^ In most previous studies,^5^–^7^ the sarcopenia status was assessed at the time of cancer diagnosis.

To the best of our knowledge, no study has clarified whether skeletal muscle change during neoadjuvant chemotherapy followed by surgery adversely affects prognosis. We hypothesized that skeletal muscle loss from pretreatment to posttreatment has a negative impact on cancer recurrence and overall survival (OS). The current study aimed to determine the relationship between skeletal muscle change and survival for patients with advanced esophageal cancer who underwent neoadjuvant chemotherapy followed by surgery.

## Patients and Methods

### Patients

**Fig. 1:**
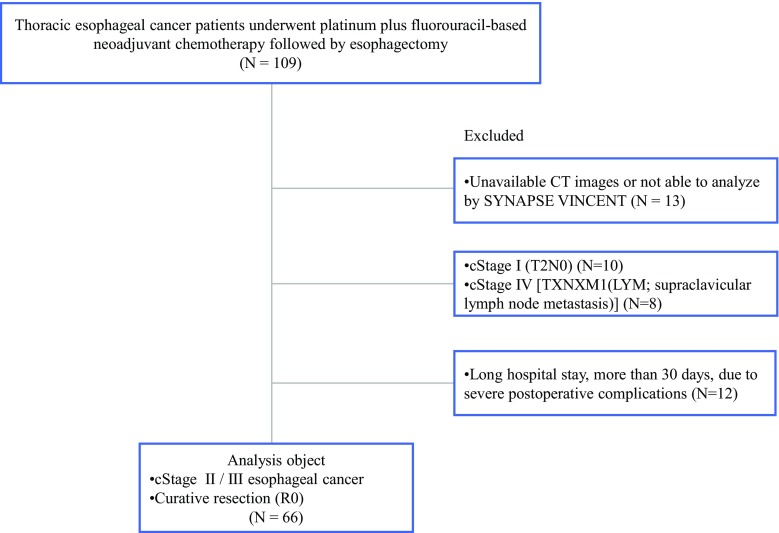
Flow diagram. The study included 66 patients with stage 2 or 3 thoracic esophageal cancer who had undergone curative resection (R0)

Between May 2004 and December 2013, 109 patients underwent platinum plus fluorouracil-based neoadjuvant chemotherapy followed by esophagectomy for thoracic advanced esophageal cancer at the Shizuoka Cancer Center Hospital (Shizuoka, Japan). The current analysis included patients who had stage 2 or 3 thoracic esophageal cancer and underwent curative resection (R0). The analysis excluded patients with a postoperative hospital stay longer than 30 days due to postoperative complications (Fig. 1).

The patients were evaluated using esophagoscopy, computed tomography (CT), and positron emission tomography (PET) before neoadjuvant chemotherapy. Clinical staging and pathologic examination of the tumors were performed according to the tumor-node-metastasis (TNM) classification, 7th edition.^18^ This study was conducted in accordance with the ethical principles based on the Declaration of Helsinki and was approved by the institutional review board of Shizuoka Cancer Center Hospital.

### Measurement of Skeletal Muscle

**Fig. 2:**
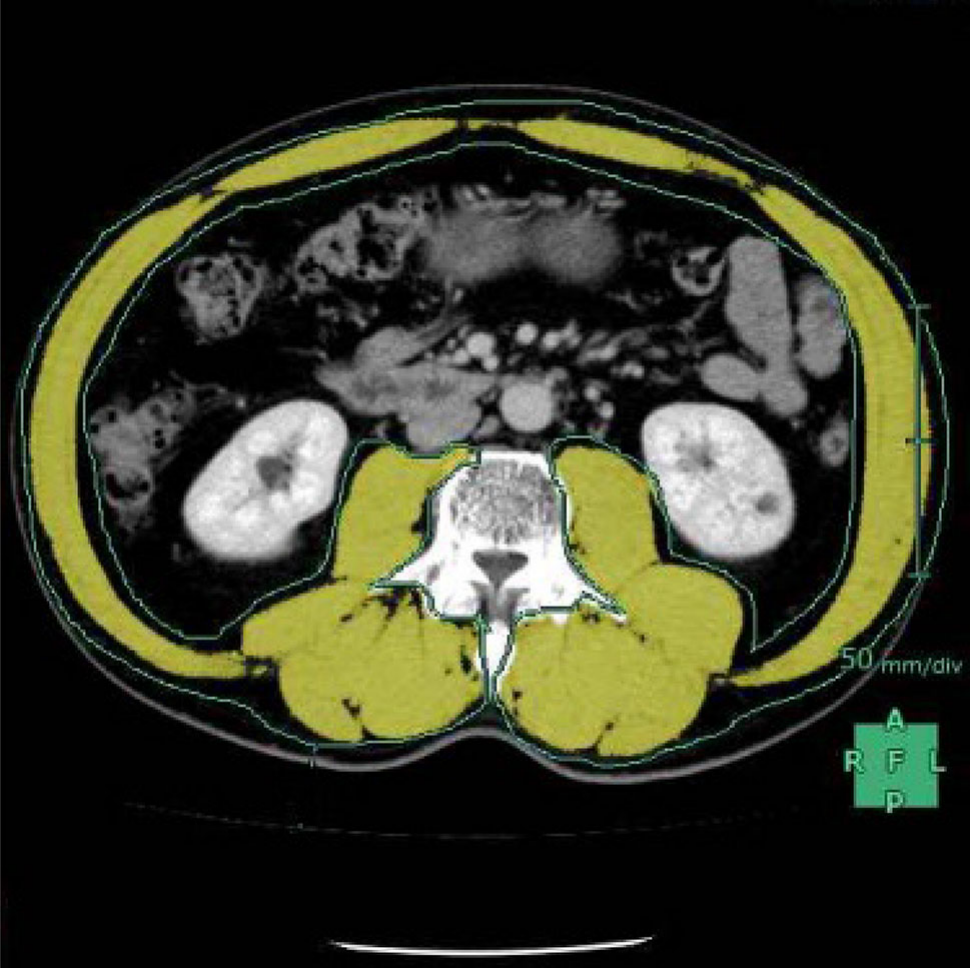
Measurement of the skeletal muscle. The total muscle cross-sectional area at the middle level of the third lumbar vertebra (L3) was measured using SYNAPSE VINCENT software. The skeletal muscle was identified and quantified using Hounsfield unit thresholds (−29 to +150) (*yellow zone*)

The total muscle cross-sectional area at the middle level of the third lumbar vertebra (L3) was measured using SYNAPSE VINCENT software (Fujifilm Co., Tokyo, Japan) before treatment and 4 months after surgery (Fig. 2). The L3 total muscle cross-sectional area was identified and quantified using Hounsfield unit thresholds (−29 to +150).^19^ The directly ascertained L3 total cross-sectional area was normalized for stature by the following calculation:

$$ {\text{L3}}\;{\text{skeletal}}\;{\text{muscle}}\;{\text{index}}\;\left( {{\text{cm}}^{2} /{\text{m}}^{2} } \right) = {\text{L3}}\;{\text{total}}\;{\text{cross-sectional}}\;{\text{area }}\left( {{\text{cm}}^{2} } \right)/{\text{height}}^{2} \left( {{\text{m}}^{2} } \right) $$

The cutoff levels of sarcopenia were 52.4 cm^2^/m^2^ for men and 38.5 cm^2^/m^2^ for women, according to a previous report.^20^

### Statistical Analysis

Clinical and pathologic variables were analyzed using the Chi square test and Fisher’s exact test. Recurrence-free survival (RFS) and OS were defined as time from 4 months after surgery (evaluation day of skeletal muscle change) to disease relapse or death from any cause, respectively. These were censored at last confirmation of survival if no events were observed until then.

Statistical analyses were performed as follows. First, we selected variables likely related to RFS and OS among all candidate clinical and pathologic variables. The univariable analyses were performed using Cox proportional hazard regression models for these variables. Due to the limited number of observed events, we selected the best model using the stepwise method so the multivariable model would secure statistical power. Second, we checked the assumption of proportionality with hazard using the Kaplan–Meier method.

All statistical analyses were performed using SPSS version 19 software (IBM Corp., Armonk, NY, USA) and R version 3.2.4. Differences were considered statistically significant at *p* values lower than 0.05.

## Results

The clinicopathologic parameters of the patients are shown in Table 1. The study included 66 patients (57 men and 9 women) with a mean age of 63.3 years. Of these 66 patients, 55 (83%) had squamous cell carcinoma, 4 (6%) had adenosquamous carcinoma, 3 (5%) had basaloid carcinoma, and 4 (6%) had adenocarcinoma. The skeletal muscle index of 55 patients (83%) met the criteria for sarcopenia.

**Table 1:** Clinicopathologic characteristics of the study patients

	*n* = 66 (*n* %)	
Age (years)	63.3 ± 8.0	
Sex (male/female)	57/9	
Anatomical subsite		
Upper third	2	3
Middle third	33	50
Lower third	31	47
Histologic type		
Squamous cell carcinoma	55	83
Adenocarcinoma	4	6
Adenosquamous carcinoma	4	6
Basaloid carcinoma	3	5
Pretreatment diagnosis (UICC 7th)		
cStage 2	27	41
cStage 3	39	59
Pathologic diagnosis (UICC 7th)		
ypStage 1	13	20
ypStage 2	16	24
ypStage 3	33	50
ypStage 4	4	6
Therapeutic effect (grade of primary tumor)		
0	11	17
1a	24	36
1b	12	18
2	17	26
3	1	2
Not evaluated	1	2
Postoperative complication ≥ Clavien-Dindo grade 2		
Anastomotic leakage	1	2
Pneumonia	6	9
Atrial fibrillation	2	3
Postoperative hospital stay (days)	16.4 ± 4.3	
Serum albumin (g/L)	44 ± 2.6	
Serum C-reactive protein (mg/dL)	0.31 ± 0.58	
BMI (kg/m^2^)	21.0 ± 2.6	
Skeletal muscle index (cm^2^/m^2^)	45.0 ± 6.5	
Pretreatment sarcopenia	55	83
Skeletal muscle change (cm^2^/m^2^)		
From baseline to post-neoadjuvant chemotherapy	+0.3 ± 3.0	
From baseline to 4 months after esophagectomy	−1.2 ± 3.7	

*UICC* Union for International Cancer Control, *BMI* body mass index

Data are presented as mean ± standard deviation or as *n* (%)

All the patients underwent platinum plus fluorouracil-based neoadjuvant chemotherapy followed by curative resection (R0). At the time of esophagectomy, the jejunostomy was created in all cases. Enteral feeding was performed from the early postoperative period and continued with oral intake at the physician’s decision to maintain the minimum energy requirement. The postoperative hospital stay was 16.4 days. The postoperative complications (Clavien-Dindo grade 2 or higher) included minor pneumonia for six patients (9%) and minor anastomotic leakage for one patient (2%). Posttreatment pathologic stage 4 disease was diagnosed for four patients (6%) because of subclavian lymphnode metastasis.

Of the 66 patients, 39 (59%) showed a skeletal muscle decrease from baseline to 4 months after esophagectomy. Body weight loss from baseline to 4 months after surgery was −10.8%. Skeletal muscle change from baseline to post-neoadjuvant chemotherapy was +0.3 ± 3.0 cm^2^/m^2^ and from baseline to 4 months after esophagectomy was −1.2 ± 3.7 cm^2^/m^2^.

During a median follow-up period of 1180 days, 13 patients had locoregional recurrence, and 13 patients had distant metastasis. The 3-year OS and RFS rates were 68.8% and 57.3%, respectively. The impacts of clinicopathologic features, including age, sex, histologic type, albumin, C-reactive protein, body mass index, pretreatment sarcopenia, weight loss rate, skeletal muscle change, and pathologic stage, on OS were evaluated using multivariable analysis (Table 2). The independent prognostic factors associated with worse OS were nonsquamous cell carcinoma subtype (hazard ratio [HR] 2.57; *p* = 0.029), posttreatment pathologic stage (HR 5.73; *p* < 0.01), and skeletal muscle wasting (HR per 1 unit decrease in skeletal muscle index, 1.16; *p* = 0.015). Additionally, posttreatment pathologic stage (HR 6.03; *p* < 0.01) and skeletal muscle wasting (HR per 1 unit decrease in skeletal muscle index, 1.11; *p* = 0.048) also were found to be independent prognostic factors associated with worse RFS. (Table 3).

**Table 2:** Uni- and multivariable Cox regression analyses for overall survival

	Univariable analysis			Multivariable analysis^a^		
	HR	*p* Value	95% CI	HR	*p* Value	95% CI
Age (≥65 years)	0.720	0.415	0.326–1.59			
Sex (male)	2.22	0.280	0.522–9.42			
Histologic type (non-SCC)	3.11	<0.01	1.337–7.232	2.57	0.029	1.10–6.02
ypStage (≥3)	4.14	<0.01	1.95–8.80	5.73	<0.01	1.94–16.7
Therapeutic effect	0.611	0.012	0.416–0.898			
Albumin	1.12	0.173	0.952–1.32			
CRP	0.213	0.136	0.028–1.63			
BMI (≥20)	0.836	0.659	0.378–1.85			
Sarcopenia	2.56	0.202	0.604–10.8			
Weight loss rate	0.937	0.052	0.877–1.00			
Skeletal muscle wasting	1.19	<0.01	1.06–1.34	1.16	0.015	1.03–1.31

*HR* hazard ratio, *CI* confidence interval, *SCC* squamous cell carcinoma, *ypStage* posttreatment pathologic stage; *CRP* C-reactive protein, *BMI* body mass index

^a^The stepwise method was performed in the multivariable analysis

**Table 3:** Uni- and multivariable Cox regression analyses for recurrence-free survival

	Univariable analysis			Multivariable analysis^a^		
	HR	*p* Value	95% CI	HR	*p* Value	95% CI
Age (≥65 years)	0.557	0.120	0.267–1.16			
Sex (male)	1.58	0.451	0.480–5.22			
Histologic type (non-SCC)	2.137	0.066	0.952–4.797			
ypStage (≥3)	6.39	<0.01	2.43–16.8	6.03	<0.01	2.29–15.9
Therapeutic effect	0.621	<0.01	0.438–0.881			
Albumin	1.09	0.221	0.950–1.25			
CRP	0.180	0.079	0.026–1.22			
BMI (≥20)	0.978	0.751	0.851–1.12			
Sarcopenia	2.06	0.236	0.625–6.77			
Weight loss rate	0.966	0.230	0.912–1.02			
Skeletal muscle wasting	1.13	0.016	1.02–1.25	1.11	0.048	1.01–1.24

*HR* hazard ratio, *CI* confidence interval, *SCC* squamous cell carcinoma, *ypStage* posttreatment pathologic stage, *CRP* C-reactive protein, *BMI* body mass index

^a^The stepwise method was performed in the multivariable analysis

## Discussion

Several retrospective studies have shown that the presence of pretreatment sarcopenia was not associated with negative short- or long-term outcomes for esophageal cancer patients who underwent neoadjuvant chemoradiotherapy^15^ or chemotherapy^5^,^7^ followed by esophagectomy. However, Sheetz et al..^6^ reported that pretreatment skeletal muscle amount was an independent predictor of both OS and disease-free survival. The prediction of OS on the basis of pretreatment sarcopenia status alone might be difficult for patients with advanced esophageal cancer, unlike patients with cancers in other parts, because skeletal muscle loss at diagnosis results not only from cancer cachexia but also from esophageal malignant stenosis. Therefore, both pretreatment sarcopenia status and postoperative change in skeletal muscle amount should be assessed.

In the field of gynecologic malignancy, reports show a relationship of skeletal muscle change during neoadjuvant chemotherapy to poor prognosis.^21^ In the cited study, the patients with advanced ovarian cancer had poor survival when skeletal muscle loss occurred during neoadjuvant chemotherapy, although a low skeletal muscle amount at pretreatment was not a prognostic factor for OS. However, it is unclear whether skeletal muscle wasting during cancer treatment is causally related to cancer recurrence and survival for patients with esophageal cancer.

Our findings indicate that skeletal muscle change from pretreatment to posttreatment might be a more important prognostic factor than pretreatment sarcopenia status. The progression of residual cancer might contribute to hypercatabolism.^22^ For postoperative patients with residual esophageal cancer, several factors, including cancer progression and nutritional deficiency, might increase the catabolic response, leading to unsustainable levels of muscle mobilization and high levels of muscle depletion because the tumor can alter energy regulation by eliciting an excessive inflammatory response. On the other hand, there is a hypothesis that skeletal muscle loss correlates with reduced physical activity, which may promote cancer recurrence. Findings have shown an association between reduced physical activity and increased cancer-specific death for postoperative patients with colon or breast cancer.^23^ Moreover, enhanced physical activity may result in benefits such as a decreased anti-inflammatory response and an improved immune function against cancer recurrence.^24^,^25^ However, it is difficult to conclude the cause and effect relation between skeletal muscle loss and cancer recurrence based on previous reports and our data. Large-scale data collection and detailed basic research are needed to assess the relationship between cancer recurrence and skeletal muscle loss.

The current study had several limitations. First, this was a single-center, retrospective study including a small number of patients. Second, selection bias may have occurred because patients with severe postoperative complications were excluded from the study. Third, the definition of sarcopenia might not have been appropriate. Most previous reports on the relationship between sarcopenic obesity and cancer^5^–^7^,^15^–^17^ have recommended sarcopenia cutoff levels of 52.4 cm^2^/m^2^ for men and 38.5 cm^2^/m^2^ for women.^20^ However, these reports have been published from Western countries, and Asian thoracic esophageal cancer patients were not included.

The proportion of patients with obesity among Asian thoracic esophageal cancer patients is small. Indeed, the mean patient body mass index (BMI) in our study was only 21 kg/m^2^, and other studies from Japan have reported a similar BMI.^26^,^27^ However, patients with adenocarcinoma were reported to have a BMI exceeding 25 kg/m^2^ in Western countries.^6^,^15^–^17^

Our findings indicated a high percentage of patients with sarcopenia (83%). However, the results associated with sarcopenia status should be interpreted with caution considering the influence of ethnic and histologic differences.

In conclusion, our findings suggest that skeletal muscle loss from baseline has a negative impact on cancer recurrence and survival. Although it is not known whether maintaining skeletal muscle amount can decrease cancer recurrence, a postoperative nutritional and physical reconditioning intervention might improve survival. We are planning a trial of prospective intervention to prevent skeletal muscle wasting in patients with esophageal cancer.
